# The Effect of Alkyl Terminal Chain Length of Schiff-Based Cyclotriphosphazene Derivatives towards Epoxy Resins on Flame Retardancy and Mechanical Properties

**DOI:** 10.3390/polym15061431

**Published:** 2023-03-14

**Authors:** Nur Atika Waldin, Zuhair Jamain

**Affiliations:** Organic Synthesis and Advanced Materials (OSAM) Research Group, Faculty of Science and Natural Resources, University Malaysia Sabah (UMS), Kota Kinabalu 88400, Sabah, Malaysia

**Keywords:** cyclotriphosphazene, Schiff-based, flame retardant, mechanical property, epoxy resin

## Abstract

A series of Schiff-based cyclotriphosphazenes with different alkyl chain length terminal ends, **4a** (dodecyl) and **4b** (tetradecyl), were synthesized and the structures were characterized using Fourier-transform infrared spectroscopy (FT-IR), and ^1^H, ^13^C, and ^31^P nuclear magnetic resonance (NMR) and carbon, hydrogen, and nitrogen (CHN) elemental analysis. The flame-retardant and mechanical properties of the epoxy resin (EP) matrix were examined. The limiting oxygen index (LOI) of **4a** (26.55%) and **4b** (26.71%) revealed a good increment compared to pure EP (22.75%). The LOI results corresponded to their thermal behavior studied using thermogravimetric analysis (TGA) and the char residue analyzed under field emission scanning electron microscopy (FESEM). The mechanical properties of EP showed a positive impact on tensile strength with a trend of EP < **4a** < **4b**. The tensile strength went from 8.06 N/mm^2^ (pure EP) to 14.36 and 20.37 N/mm^2^, indicating that the additives were compatible with epoxy resin.

## 1. Introduction

Being fire retardant is one of the main concerns regarding fire safety. A fire retardant is a substance that is used to slow down the spread of fire by eliminating the cycle of fuel. In other words, this means reducing or isolating the source of fire such as combustible gas, heat and oxygen. The involvement of synthetic chemistry in manufacturing to improve polymer’s properties has become of great importance to fire safety.

Epoxy resin (EP) had already gain attention due to its outstanding characteristics and wide application as an adhesive, flooring/automotive coating as well as in electronic devices and construction as an asphalt/concrete mixture [1,2]. In general, an unmodified polymer material such as EP is flammable because it is made up by hydrocarbons chains and easily burns rapidly when exposed to fire; thus, it still cannot meet the requirements of many industrial fields [3,4]. Therefore, as a way to improve its properties, halogen-containing flame retardants (FR) were widely incorporated into this matrix, using either reactives or additives. Unfortunately, halogenated FR, such as tetrabromobisphenol A (TBBPA), [tris(1,3-dichloro-2-propyl) phosphate] (TDCPP), chlorocyclotriphosphazene (HCCP) and more, have become widespread global contamination issues, especially to the environment and human health [5,6,7,8].

It has been proven that the effects of various modifying additives and fillers on the flammability reduction, and physical and chemical properties of EP composites, is determined by some factors. A good fire resistance of EP was affected by the protective layer formed on the surface which acts as a physical barrier during combustion. This can be achieved as the yield of carbonized structures increase in the presence of phosphorus [9,10,11]. Additionally, the content of nitrogen is considered as the main factor in designing a high-efficiency flame retardant EP. Ammonia was released during combustion which blocked the entry of air and broke the cycle of fire [12].

At present, an approach to overcome these issues is using alternative free-halogen FR such as hexasubstituted cyclotriphosphazene derived from a well-known FR agent, which is HCCP [13,14,15]. HCCP is commonly used as starting material for the synthesis, because the presence of a reactive P-Cl bond makes it eligible to undergo nucleophilic substitution [16]. In addition, the content of phosphorus, acting as a radical scavenger, and nitrogen, which is responsible for being a charring agent by releasing inert gaseous by-products during combustion, can greatly enhance the flame resistance of a material [17,18].

The authentic characteristics of cyclotriphosphazene have become more fascinating and varied substituent groups could be explored with desirable applications in industry, such as being a flame retardant, in nanomaterials, biomedicine and more [19,20,21]. Interestingly, the properties of this compound can be functionalized depending on the nature of the substituents attached to the core compound [22]. The incorporation of the substituent into the core compound usually affects their compatibility when added into a polymer. As such, more studies on improving ways of introducing flame-retardant additives without failure is highly worthwhile. For example, Zhang et al. synthesized hexaphenoxycyclotriphosphazene (HPCTP) with different weight percentages, which provided excellent flame resistance to EP [23]. Qu et al. also improved the flame-retardant properties of EP by synthesizing capsulated polymethyl methacrylate (PMM) with a HPCTP additive [24]. Another study by Tarasov et al. successfully synthesized a phosphazene-containing EP based on Bisphenol F, which had a positive impact on its flame-retardant properties, yet maintained its mechanical properties with a low phosphazene fraction [25].

Studies related to Schiff-based compounds were reported showing a wide range of applications, especially as a flame retardant [26,27,28,29]. The presence of a Schiff-based moiety was characterized using FT-IR to show the functional group of C=N at the range of 1600–1610 cm^−1^ and NMR showed the proton -N=CH- downfield [30]. The purity content of carbon and nitrogen in C=N was further confirmed using CHN elemental analysis with a convincing percentage error of calculated and found values below 4%. A functioning Schiff-based compound effectively improved the fire resistance of a polymer matrix by forming a continuous and compact char layer [28].

In this research, two Schiff-based compounds bridged by hydrazine, with different length alkyl chain end terminals attached to cyclotriphosphazene as a substituent, were synthesized in order to studied their effect on the flame retardant and mechanical behaviors of epoxy resin. This study focuses on achieving the highest fire-retardant properties with fewer additives used, which is a 1wt% fire-retardant compound as an additive. In this contribution, the Schiff-based compound is expected to encourage the char-forming ability by maintaining the linearity and forming a stable cross-link, which is also expected to enhance flame resistance [31,32]. The different length alkyl chain (dodecyl and tetradecyl) was taken into consideration as it facilitates the toughening of the epoxy resin, contributing to a good mechanical property [33].

## 2. Experimental

### 2.1. Chemicals

The chemicals and solvents that were used in this research were 4-hydroxybenzaldehyde, 1-bromododecane, 1-bromotetradecane, *N*,*N*-dimethylformamide (DMF), dichloromethane (DCM), hydrazine sulfate, phosphonitrilic chloride trimer (HCCP), potassium carbonate, potassium iodide, methanol, ethanol, acetone, glacial acetic acid, triethylamine, and epoxy resin/amine. All these chemicals and solvents were used without purification and purchased from Merck (Darmstadt, Germany), Qrëc (Asia) (Selangor, Malaysia), Sigma-Aldrich (Steinheim, Germany), Acros Organics (Geel, Belgium), and BDH (Dubai, UAE). Table 1 shows the typical properties of epoxy resin and HCCP.

### 2.2. Syntheses

#### 2.2.1. Synthesis of 4-alkoxybenzaldehyde, **1a**–**b**

Both 0.1. mol 4-hydroxybenzaldehyde and 0.1 mol 1-bromodecane were dissolved separately in 20 mL of *N*,*N*-dimethylformamide, DMF, and mixed into a 250 mL round-bottom flask. Added to the mixture were 0.15 mol Potassium carbonate, K_2_CO_3_, and 0.1 mol potassium iodide, KI, and it was then refluxed for 12 h. The reaction progress was monitored using a thin-layer chromatograph (TLC). Upon completion, the mixture was poured into 500 mL of cold water and extracted using dichloromethane (DCM). The organic layers were collected and dried in anhydrous sodium sulphate to remove excess water. The product was filtered and evaporated overnight to fully removed access solvents. The same method was used to synthesize compound **1b**.

4-dodecyloxybenzaldehyde, **1a**:

Yield: 23.66 g (81.45%), light-yellow oil. FTIR (cm^−1^) 2922 and 2852 (Csp^3^-H stretching), 2732 (C-H aldehyde stretching), 1689 (C=O stretching), 1599 (C=C stretching), 1253 (C-O stretching). ^1^H-NMR (600 Hz, CDCl3) δ, ppm: 9.76 (s, 1H), 7.72 (d, *J* = 4.8 Hz, 2H), 6.90 (d, *J* = 8.94 Hz, 2H), 3.93 (t, *J* = 6.18 Hz, 2H), 1.71 (m, *J* = 8.94 Hz, 2H), 1.38–1.18 (m, *J* = 7.56 Hz, 18H), 0.80 (t, *J* = 6.9 Hz, 3H). ^13^C-NMR (150 Hz, CDCl3) δ, ppm: 190.27, 164.12, 131.73, 129.69, 68.25, 31.11, 29.61, 29.57, 29.53, 29.32, 29.00, 26.19, 25.91, 22.63.

4-tetradecyloxybenzaldehyde, **1b**:

Yield: 29.91 g (93.90%), white powder. FTIR (cm^−1^) 2914 and 2847 (Csp^3^-H stretching), 2733 (C-H aldehyde stretching), 1687 (C=O stretching), 1599 (C=C stretching), 1250 (C-O stretching). 1H-NMR (500 Hz, CDCl3) δ, ppm: 9.85 (s, 1H), 7.80 (d, *J* = 5.0 Hz, 2H), 6.97 (d, *J* = 5.0 Hz, 2H), 4.01 (t, *J* = 5.0 Hz, 2H), 1.81–1.76 (m, *J* = 5.0 Hz, 2H), 1.57–1.41 (m, *J* = 5.0 Hz, 2H), 1.36–1.24 (m, *J* = 5.0 Hz, 20H), 0.87 (t, *J* = 5.0 Hz, 3H). ^13^C-NMR (125 Hz, CDCl3) δ, ppm: 190.76, 164.29, 131.97, 129.79, 68.45, 31.91, 29.68, 29.66, 29.64, 29. 57, 29.53, 29.34, 29.33, 29.06, 25.96, 22.67. 14.09 CHN elemental analysis: Calculated for C_21_H_34_O_2_: C: 79.19%, H: 10.76%; Found: C: 78.84%, H: 10.67%.

#### 2.2.2. Synthesis of 4-(substituted benzylidene)hydrazinium, ****2a**–**b****

0.008 mol of finely powdered hydrazine sulphate was suspended in 20 mL of hot distilled water and then 10 mol of sodium acetate anhydrous was added to the suspension. The mixture was boiled for 5 min and stirred until fully dissolved. Then, the mixture was allowed to cool down to 50 °C before 20 mL of hot ethanol was added; a cloudy solution started to appear. This mixture was filtered, and the filtrate was used for the next step. In a 100 mL round-bottom flask, the filtrate was added dropwise to 0.008 mol of 4-dodecyloxy benzaldehyde, **1a**, and stirred for 12 h at room temperature. TLC was used to monitor the reaction progress. The precipitate obtained was filtered and dried. The crude product was then recrystallized from acetone. The same method was used to synthesize compound **2b**.

4-Dodecyloxybenzylidene hydrazinium, **2a**:

Yield: 2.26 g (90.94%), light-yellow powder. FTIR (cm^−1^) 2916 and 2848 (Csp^3^-H stretching), 1604 (C=N stretching), 1509 (C=C stretching), 1172 (C-O stretching). ^1^H-NMR (500 Hz, DMSO-d_6_) δ, ppm: 8.47 (s, 1H), 7.79 (d, *J* = 5.00 Hz, 2H), 6.64 (d, *J* = 5.00 Hz, 2H), 4.06 (t, *J* = 5.00 Hz, 2H), 1.78–1.72 (m, *J* = 10.00 Hz, 2H), 1.46–1.26 (m, *J* = 5.00 Hz, 18H), 0.88 (t, *J* = 10.00 Hz, 3H). ^13^C-NMR (125 MHz, DMSO-_6_) δ, ppm: 161.32, 147.55, 141.36, 130.01, 68.57, 31.64, 29.35, 29.33, 29.31, 29.29, 29.15, 29.08, 28.99, 25.88, 25.35, 22.35, 14.07. CHN elemental analysis: Calculated for C_19_H_32_N_2_O: C: 74.95%, H: 10.59%, N: 9.20%; Found: C: 74.83%, H: 10.47%, N: 9.11%.

4-Tetradecyloxybenzylidene hydrazinium, **2b**:

Yield: 2.43 g (89.94%), light-yellow powder. FTIR (cm^−1^) 2915 and 2848 (C-H sp3 stretching), 1604 (C=N stretching), 1509 (C=C stretching), 1172 (C-O stretching). ^1^H-NMR (500 Hz, DMSO-d_6_) δ, ppm: 8.47 (s, 1H), 7.79 (d, *J* = 5.00 Hz, 2H), 6.59 (d, *J* = 10.00 Hz, 2H), 4.02 (t, *J* = 10.00 Hz, 2H), 1.73–1.67 (m, *J* = 10.00 Hz, 2H), 1.42–1.22 (m, *J* = 5.00 Hz, 22H), 0.83 (t, *J* = 5.00 Hz, 3H). ^13^C-NMR (125 MHz, DMSO-_6_) δ, ppm: 161.32, 147.55, 141.36, 130.01, 68.57, 31.64, 29.36, 29.35, 29.34, 29.33, 29.30, 29.29, 29.15, 19.08, 28.99, 25.88, 22.35, 14.07. CHN elemental analysis: Calculated for C_21_H_36_N_2_O: C: 75.85%, H: 10.919%, N: 8.42%; Found: C: 75.79%, H: 10.86%, N: 8.38%.

#### 2.2.3. Synthesis of hexakis(4-formlyphenoxy)cyclotriphosphazene, **3**

4-hydroxybenzaldehyde (0.08 mol) and triethylamine (0.1 mol) was dissolved in 150 mL of acetone and the mixture was cooled to 0 °C in an ice bath. The mixture was then stirred for 30 min. A solution of hexachlorocyclotriphosphazene, HCCP, was prepared by dissolving 0.01 mol of HCCP into 50 mL of acetone, and was added dropwise into the mixture. The temperature was maintained at 0 °C for 2 h. Then, the mixture was allowed to reach room temperature and was continuously stirred for an additional 94 h. Upon completion, the mixture was monitored using TLC. When the reaction is completed, 250 mL of cold water was poured into the mixture and left overnight in the fridge. The precipitate was filtered and washed with water and left to dry. The precipitate was recrystallized from methanol.

Hexakis(4-formlyphenoxy)cyclotriphosphazene, **3**:

Yield: 3.99 g (92.71%), white powder. FTIR (cm^−1^): 2729 (H-C=O), 1697 (C=O stretching), 1595 (C=C stretching), 1204 (C-O stretching), 1150 (P=N stretching), 950 (P-O-C stretching). ^1^H-NMR (500 MHz, DMSO-d_6_) δ, ppm: 9.90 (s, 1H), 7.78 (d, *J* = 10.00 Hz, 2H), 7.16 (d, *J* = 10.00 Hz, 2H). ^13^C-NMR (150 MHz, DMSO-d_6_) δ, ppm: 191.69, 153.59, 133.55, 131.46, 121.03. ^31^P-NMR (500 MHz, DMSO-d_6_) δ, ppm: 7.60. CHN elemental analysis: Calculated for C_42_H_30_N_3_O_12_P_3_: C: 58.55%, H: 3.51%, N: 4.88%; Found: C: 58.30%, H: 3.45%, N: 4.80%.

#### 2.2.4. Synthesis of hexakis{4-((E)-((4-((E)-4-substituted-benzylidene)hydrazine-1-ylidene)methyl)phenoxy}triazophosphazene, ****4a**–**b****

A mixture with a ratio of 1:8 hexasubstituted cyclotriphosphazene (0.58 mol), **3** and intermediate **2a** were mixed with 40 mL of methanol in a 250 mL round-bottom flask. A few drops of glacial acetic acid were added into the mixture as a catalyst, and stirred for 48 h at room temperature. TLC was used to monitor the reaction progress. Upon completion, the mixture was cooled in ice water and the precipitate formed was filtered and dried. The crude product was recrystallized from methanol. The same procedure was used to synthesize **4b**.

Hexakis{4-((E)-((4-((E)-4-dodecyloxy-benzylidene)hydrazine-1 ylidene)methyl)phenoxy} triazophosphazene, **4a**:

Yield: 1.35 g (90.00%), light-yellow powder. FTIR (cm^−1^): 2916 and 2848 (Csp^3^-H stretching), 1603 (C=N stretching), 1508 (C=C stretching), 1249 (C-O stretching), 1173 (P=N stretching), 959 (P-O-C stretching). ^1^H-NMR (500 Hz, DMSO-d_6_) δ, ppm: 8.67 (s, 1H), 8.56 (s, 1H), 7.96 (d, *J* = 5.00 Hz, 2H), 7.88 (d, *J* = 10.00 Hz, 2H), 7.57 (d, *J* = 10.00 Hz, 2H), 7.05 (d, *J* = 5.00 Hz, 2H), 4.08 (t, *J* = 10.00 Hz, 2H), 1.79-1.73 (q, *J* = 5.00 Hz, 2H), 1.47–1.30 (m, *J* = 5.00 Hz, 18H), 0.88 (t, *J* = 5.00 Hz, 3H). ^13^C-NMR (150 MHz, DMSO-d_6_) δ, ppm: 162.09, 162.03, 159.48, 149.82, 149.15, 136.57, 130.56, 129.31, 122.34, 115.49, 68.64, 31.64, 29.31, 29.26, 29.13, 19.12, 29.09, 28.97, 25.88, 22.35, 14.08. ^31^P-NMR (500 MHz, DMSO-d_6_) δ, ppm: 8.50. CHN elemental analysis: Calculated for C_156_H_210_N_15_O_12_P_3_: C: 72.61%, H: 8.20%, N: 8.14%; Found: C: 72.55%, H: 8.16%, N: 8.09%.

Hexakis{4-((E)-((4-((E)-4-tetradecyloxy-benzylidene)hydrazine-1 ylidene)methyl)phenoxy} triazophosphazene, **4b**:

Yield: 1.41 g (88.68%), light-yellow powder. FTIR (cm^−1^): 2916 and 2848 (Csp^3^-H stretching), 1603 (C=N stretching), 1509 (C=C stretching), 1250 (C-O stretching), 1173 (P=N stretching), 959 (P-O-C stretching). ^1^H-NMR (500 Hz, DMSO-d_6_) δ, ppm: 8.66 (s, 1H), 8.54 (s, 1H), 7.96 (d, *J* = 10.00 Hz, 2H), 7.87 (d, *J* = 10.00 Hz, 2H), 7.55 (d, *J* = 10.00 Hz, 2H), 7.04 (d, *J* = 10.00 Hz, 2H), 4.08 (t, *J* = 5.00 Hz, 2H), 1.79–1.73 (q, *J* = 5.00 Hz, 2H), 1.48–1.30 (m, *J* = 5.00 Hz, 22H), 0.88 (t, *J* = 10.00 Hz, 3H). ^13^C-NMR (150 MHz, DMSO-d_6_) δ, ppm: 162.18, 162.14, 159.33, 150.70, 150.04, 136.54, 130.54, 129.28, 122.30, 115.56, 68.77, 31.68, 29.38, 29.34, 29.20, 29.18, 29.13, 29.12, 29.02, 29.00, 25.92, 22.34, 14.01. ^31^P-NMR (500 MHz, DMSO-d_6_) δ, ppm: 8.52. CHN elemental analysis: Calculated for C_168_H_234_N_15_O_12_P_3_: C: 73.41%, H: 8.58%, N: 7.64%; Found: C: 73.38%, H: 8.54%, N: 7.61%.

#### 2.2.5. Preparation of Molded Epoxy Resin with Compounds **4a** and **4b**

The same method was used to prepare the epoxy matrix for the flame retardant and mechanical properties test. The molded EP was prepared by blending 1 wt.% of the final compounds, **4a** and **4b**, with epoxy resin and mixing it until the compound was completely dissolved. DDM was then added into the mixture with a 1:3 ratio, and stirred until it was homogenous. The mixture was poured into a silicon mold and cured at 30 °C for 24 h. The molding dimension for fire retardant (LOI test) and mechanical testing was according to BS 2782: Part 1: Method 141, and ISO 4589 and ASTM D638, respectively. Three replicates of each additive were prepared for the LOI test, with five replicates for the mechanical test.

### 2.3. Measurement and Characterization

The structure of all the synthesized intermediates and final compounds were characterized using spectroscopy analysis. The functional group was detected on a Fourier-transform infra-red (FT-IR) spectroscopy Bruker Alpha II (Platinum ATR) over the wavenumber range 500–4000 cm^−1^. ^1^H, ^13^C, and ^31^P nuclear magnetic resonance (NMR) spectra were obtained by a Bruker 500 MHz Ultrashield spectrometer (Bruker, Coventry, UK) using CDCl_3_ and DMSO as a solvent system. Carbon, hydrogen and nitrogen (CHN) elemental analysis was used to compare the theoretical and laboratory values of carbon, hydrogen and nitrogen of the compounds (PerkinElmer, Waltham, MA, USA).

For thermal analysis, thermogravimetric analysis (TGA) was performed on a TGA/DSC (Mettler Toledo, Selangor, Malaysia) from 50–600 °C at heating rate of 10 °C min^−1^ in N_2_ atmospheres. The limiting oxygen index (LOI) values were measured using S.S Instruments Pvt. Ltd. (Delhi, India) in accordance with BS 2782: Part 1: Method 141 and ISO 4589 with a dimension of 120 mm × 10 mm × 4 mm. Three specimens for each sample were tested and the average value was reported. Field emission scanning electron microscopy (FESEM) was conducted using JEOL JSM-7900F (Petaling Jaya, Malaysia) magnified 5000 times.

The mechanical behavior was determined according to tensile strength using a universal tensile machine (UTM) GOTECH/AI-7000L-10 (Taichung City, Taiwan). The dumbbell-shaped tensile sample was measured according to ASTM D638. Five specimens for each sample were tested and the average value was reported.

## 3. Results and Discussion

### 3.1. Synthesis of the Intermediates and Final Compounds

**Scheme 1:**
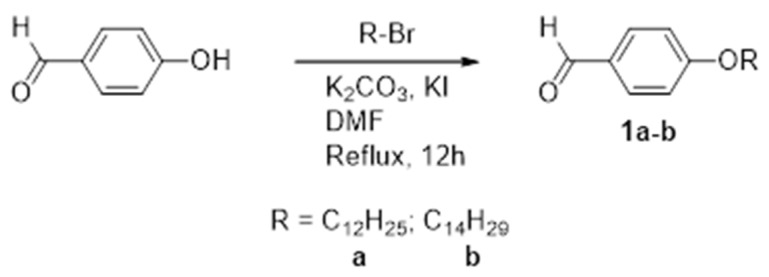
Alkylation reaction of intermediates **1a**–**b**.

**Scheme 2:**
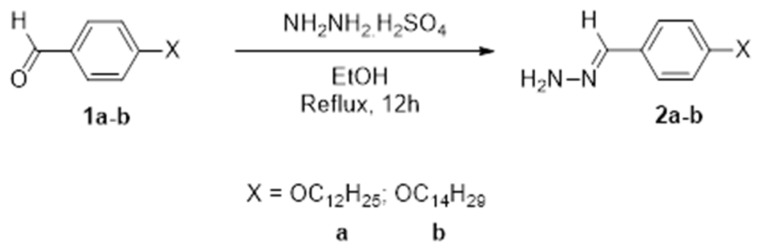
Condensation reaction of intermediates ****2a**–**b****.

**Scheme 3:**
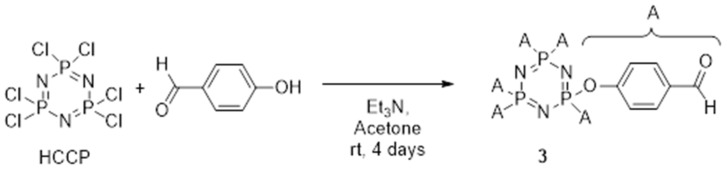
Synthesis of intermediate **3**.

**Scheme 4:**
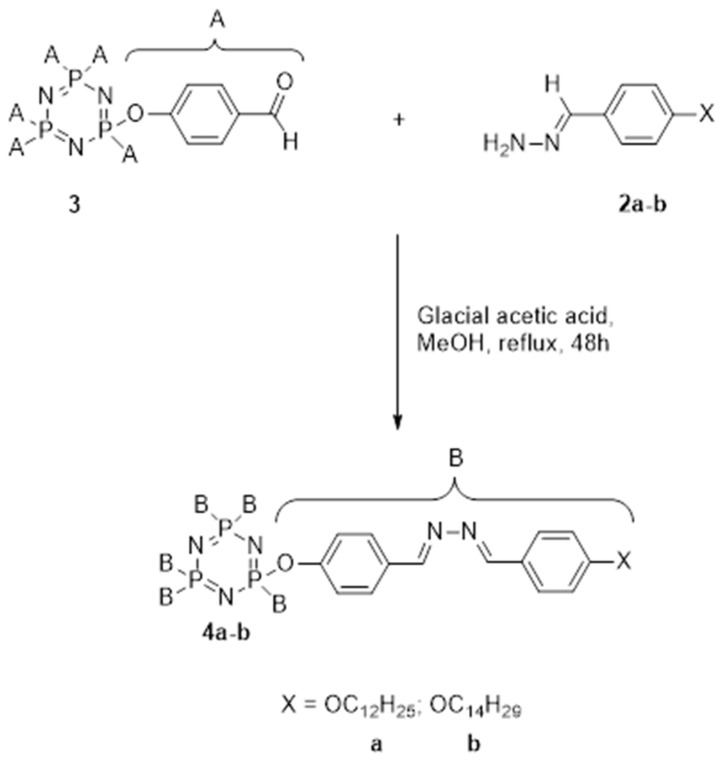
Synthesis of hexasubstituted cyclotriphosphazene compounds, ****4a**–**b****.

In this study, a new hexasubstituted cyclotriphosphazene has been synthesized and characterized. Intermediates **1a**–**b** were synthesized using an alkylation reaction of 4-hydroxybenzaldehyde with different alkyl chains (dodecyl and tetradecyl) (Scheme 1) [35]. Intermediates ****2a**–**b**** were synthesized using a condensation reaction of **1a-c** with hydrazinium sulphate [36]. The reaction of hexachlorocyclotriphospazene, HCCP, with 4-hydroxybenzaldehyde gave intermediate **3** [37] and further reacted with intermediates ****2a**–**b**** to formed final compounds ****4a**–**b**** [35]. The overall synthesis pathway for hexasubstituted cyclotriphosphazene compounds with different alkyl chain terminal ends with hydrazine bridged is shown in Scheme 1, Scheme 2, Scheme 3 and Scheme 4.

All intermediates and final compounds were characterized using spectroscopic analysis: FT-IR, NMR, and CHN elemental analysis. In addition, the effects of hexasubstituted cyclotriphosphazene as a fire retardant was studied using TGA and LOI, and the surface morphology of char residue was investigated under FESEM. The tensile strength was used to determine the mechanical behavior of the final compounds.

### 3.2. Characterization of Chemical Structure

#### 3.2.1. Fourier-Transform Infrared (FT-IR) Spectral Analysis

**Figure 1:**
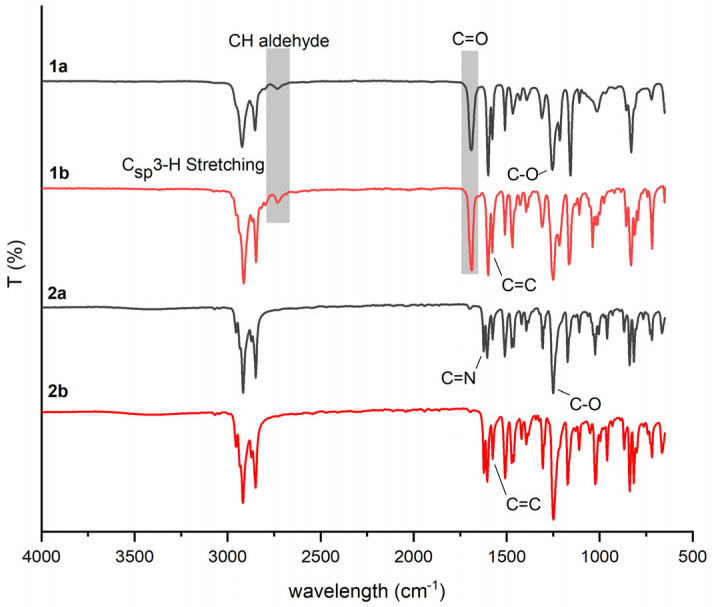
Overlay of FT-IR spectra of compounds **1a**–**b** and ****2a**–**b****.

**Figure 2:**
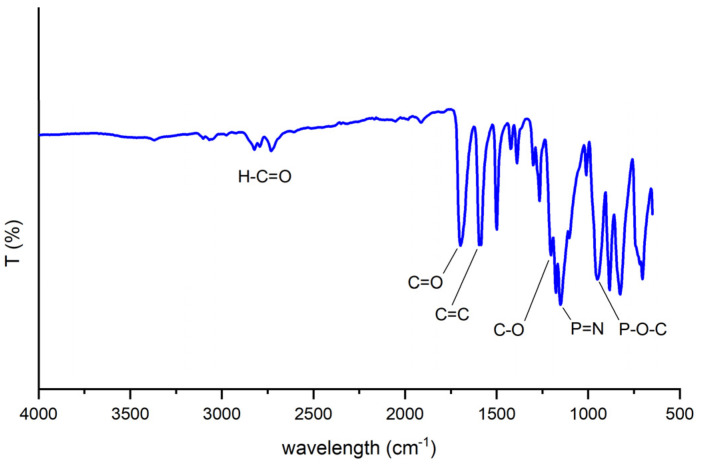
FT-IR spectrum of compound **3**.

**Figure 3:**
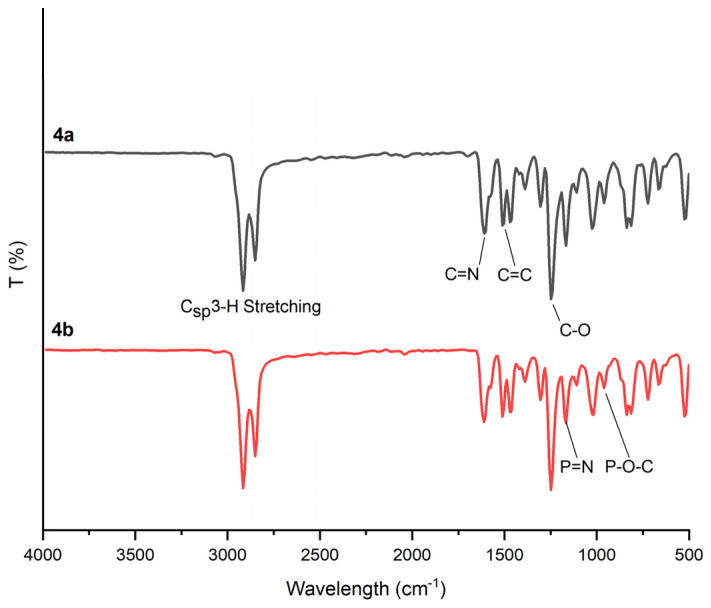
Overlay of FT-IR spectra of compounds ****4a**–**b****.

The infrared spectra of all the compounds, **1a**–**b**, ****2a**–**b****, **3**, and ****4a**–**b**** were measured using FT-IR spectrometry and data were illustrated in Figure 1, Figure 2 and Figure 3. Figure 1 presents the FT-IR spectra of compounds, **1a**–**b** and ****2a**–**b****. The absorption existing at 2922 and 2852 cm^−1^ was attributed to C_sp3_-H stretching. The C-H aldehyde can be seen at 2732 cm^−1^, and at peak 1689 cm^−1^, it corresponds to C=O stretching for compounds **1a**–**b**. Moreover, the disappearance of these peaks for ****2a**–**b**** confirmed the condensation reaction had been successfully completed. In addition, the adsorption at 1599 cm^−1^ and a sharp peak at 1253 cm^−1^ was assigned to C=C on an aromatic ring and C-O stretching, respectively. The characteristics of the Schiff-based compound was attributed to the appearance of a C=N peak at 1604 cm^−1^.

The FT-IR spectrum of compound **3** is shown in Figure 2. The characteristic of hexakis(4-formlyphenoxy)cyclotriphosphazene can be observed at 2729 cm^−1^ for H-C=O. The peaks at 1697 and 1595 cm^−1^ were assigned to C=O and C=C stretching for the aromatic ring, respectively, while the peak at 1204 cm^−1^ corresponded to the adsorption peak of C-O. The cyclotriphosphazene peak attached to the compound can be seen at the peak 1150 cm^−1^, associated with the P=N ring, and 950 cm^−1^ for the P-O-C stretching vibration of aromatic groups connected to the cyclic phosphorus atoms.

The FT-IR spectra for the final compounds, **4a** and **4b**, are presented in Figure 3. As seen in Figure 3, a similar peak pattern corresponding to C-H stretching, appeared at 2867 and 2929 cm^−1^. In addition, the C=N stretching for the Schiff-based linkage appeared at 1603 cm^−1^. Moreover, C=C stretching for the aromatic ring and C-O stretching are shown at 1509 cm^−1^ and 1249 cm^−1^, respectively. For cyclotriphophazene, the P=N ring was assigned at 1173 cm^−1^, with P-O-C bending at 959 cm^−1^.

#### 3.2.2. Nuclear Magnetic Resonance (NMR) Characterization of Final Compound

**Figure 4:**
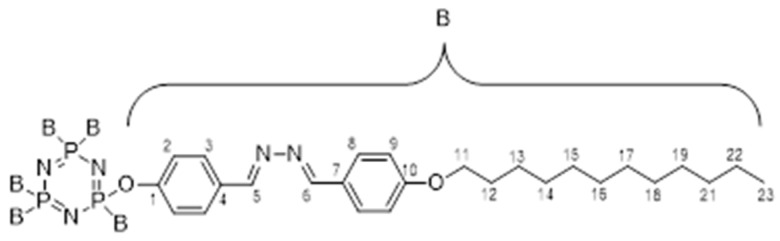
Structure of compound **4a** with complete atomic numbering.

Compound **4a** was used as a representative structure for the other compounds. The structure of compound **4a** with complete atomic numbering is shown in Figure 4.

**Figure 5:**
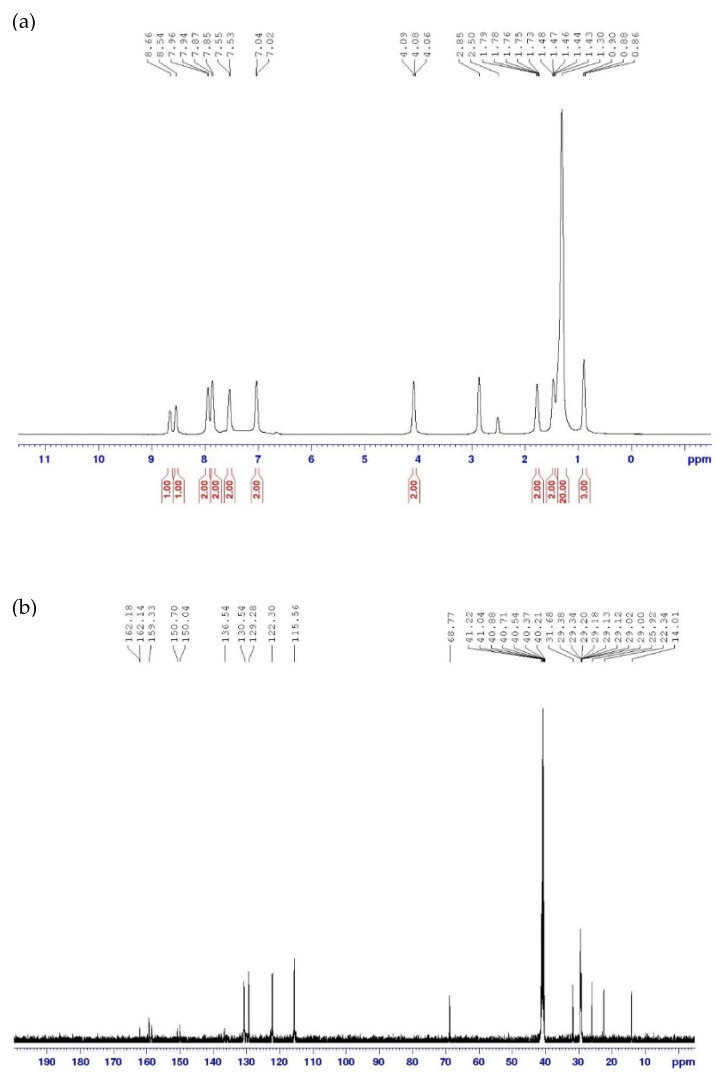

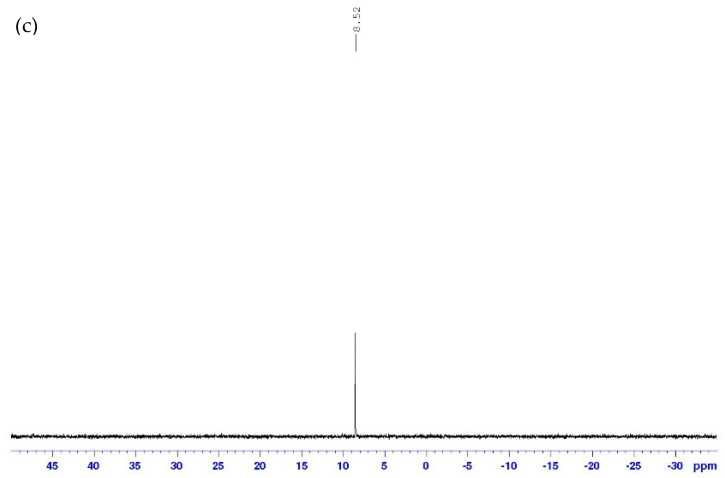
(**a**) ^1^H NMR, (**b**) ^13^C NMR, and (**c**) ^31^P NMR spectra of compound **4a**.

Figure 5a shows the ^1^H-NMR spectrum of compound **4a**. Two singlets downfield at δ 8.67 and 8.56 ppm were ascribed to two azomethine proton signals. The doublets at δ 7.96, 7.88, 7.57, and 7.05 ppm were assigned to the eight protons from two aromatic rings. The triplets in the upfield region of δ 4.08 ppm corresponded to an aliphatic chain and another methylene appeared from δ 0.89 to 1.79 ppm.

Figure 5b shows the ^13^C NMR spectrum of compound **4a**. The total carbon presence in the compound corresponding with the peaks shown in the spectrum indicated that **4a** has 23 carbons in its side arms. These consist of two azomethine, four aromatic, four quaternary, 11 methylene, and one methyl carbon. Figure 5c shows the ^31^P NMR spectrum of compound **4a**. The peak at 8.52 ppm supported the hexa-functionality in the chemical structure of the phosphorus atom in compound **4a**.

#### 3.2.3. CHN Elemental Analysis

All compounds were fully characterized using CHN elemental. The content of C, H, and N atoms listed in Table 2 fitted well with those calculated according to their molecular structures in Scheme 1, Scheme 2, Scheme 3 and Scheme 4. The percentage error was calculated and proved the purity, as the percentage error was below 2% for all compounds. Elemental analysis provides essential information about the elemental composition and guarantees the sufficient purity of a substance [38].

### 3.3. Effect of Hexasusbstituted Cyclotriphosphazene Derivatives on the Flame Retardancy

#### 3.3.1. Thermogravimetric Analysis of Final Compounds

Thermal behavior of composite materials was analyzed using thermogravimetric analysis (TGA). It has an important role in the study of flame-retardant composites, as it provides important information for evaluation through the thermal decomposition profile [39].

**Figure 6:**
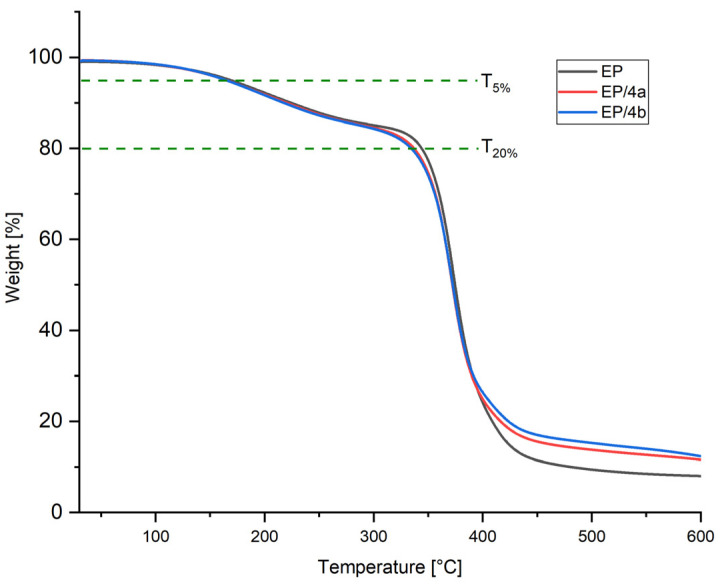
TGA curves of EP and EP incorporated with compounds **4a** and **4b**.

**Figure 7:**
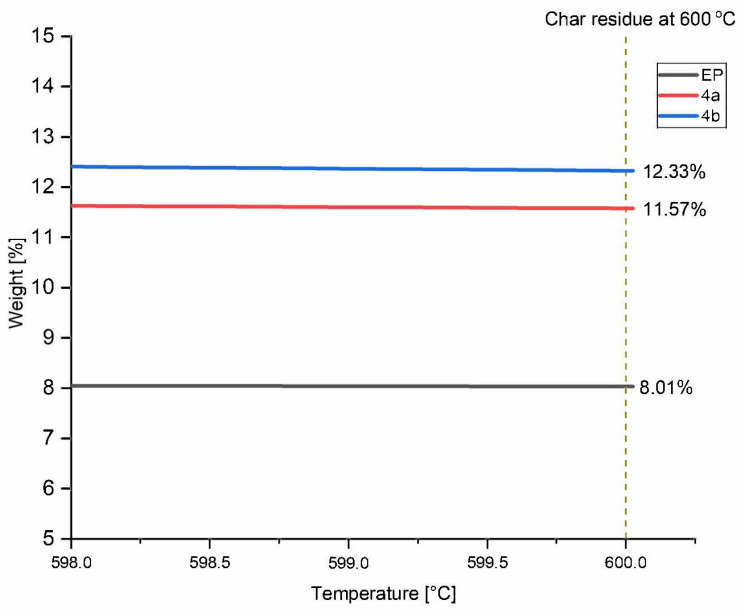
Char residue curve for EP, **4a** and **4b**.

**Figure 8:**
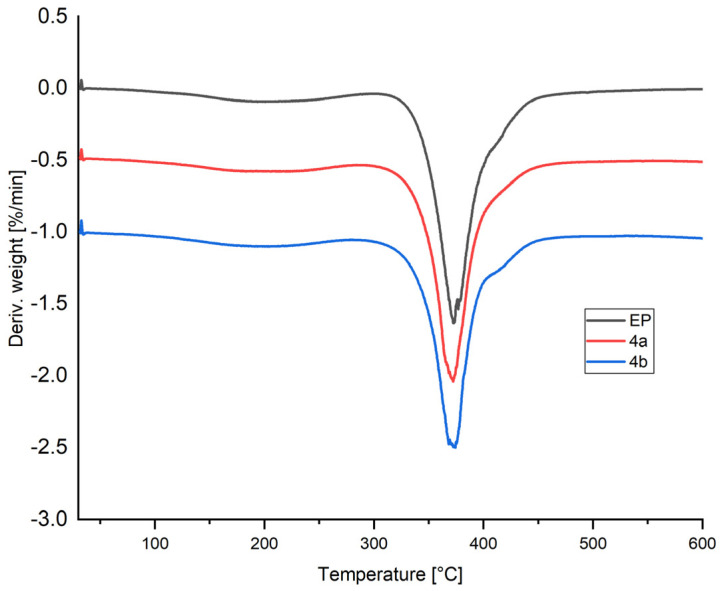
DTG curves of EP, **4a** and **4b**.

The information related to TGA is shown in Figure 6 and the detailed data are tabulated in Table 3. Other related information on char residue and a derivative thermogravimetry (DTG) graph are shown in Figure 7 and Figure 8, respectively. The thermal stability of the samples was evaluated under nitrogen atmospheres, and showed a two-stage degradation process, which was attributed to the rearrangement of the EP structure with the additives and the decomposition of EP [40].

As shown in Figure 6, a slow mass loss take place which defined the decomposition temperature at 5% (T_5wt%_) and 20% (T_20wt%_) for all compounds. It can be seen that EP with flame-retardant (FR) additive compounds **4a** and **4b** has a much lower thermal decomposition than that of pure EP. Compared with T_20wt%_ of EP with FR additives, the temperature dropped from 344.6 °C (pure EP) to 337.9 and 334.5 °C. This might be due to the contribution of the synergetic FR additives in the EP. The P-N content decomposes at a lower temperature allowing the carbonization of the EP matrix [41,42].

On the other hand, the DTG graph shows that the T_max_ decomposition of EP incorporated with FR additives shows a contradictory where a slight increment can be seen (Figure 8). This might be due to the increasing thermal degradation activation energy of the matrix, which therefore enhanced the stability of the matrix [41,43]. It is noteworthy that the incorporation of FR additives into the EP showed an effect of promoting the char residue yield at 600 °C. The char yield was from 8.0 to 11.6% when compound **4a** was added and increased to 12.3% when compound **4b** was added, indicating a strong catalyzing carbonization and deceleration of the heat transfer by providing a physical barrier.

Besides the well-known P-N which provides a synergetic effect to enhance the FR of a matrix, the Schiff-based compound attached to the system improved the stability of the FR compounds. It is also noticeable that the alkyl chain contributes to the promotion of thermal stability. Thus, this leads an enhanced thermal resistance of the EP.

#### 3.3.2. Limiting Oxygen Index (LOI) Test

The fire-retardancy for all intermediates, ****2a**–**b****, **3**, and the final compounds of hexasubstituted cyclotriphosphazene, ****4a**–**b****, was measured using the LOI test, as summarized in Table 4.

It can be seen that the LOI values of EP incorporated with non-halogenated cyclotriphosphazene (ratio EP 1:3 with 1wt.% additive) showed a significant increase from 22.75 (pure EP) to 26.55% when compound **4a** with alkyl group dodecyl was incorporated, and keep increasing to 26.71% when compound **4b** with alkyl group tetradecyl was added. This result can be related to the Schiff-based linkage and the alkyl chain length attached to the P-N system bringing a better fire-retardant performance. In addition, the effect of hexa-functionality from cyclotriphosphazene with a high content of phosphorus and nitrogen is clear, as it is a well-known compound with a high thermal stability [35,44].

A better insight into the Schiff-based compound and hydrazine bridge as well as alkyl group chain terminal end effects on EP was also sought, by incorporating 1wt.% of 4-(substitutedbenzylidene)hydrazinium, ****2a**–**b****, with 1:3 EP. A slight increase in the LOI value can be seen, with 23.42 and 23.53% for dodecyl and tetradecyl, respectively. This behavior can be explained by the Schiff-based compound and hydrazine bridge forming stable cross-linked structures, which were responsible for the charring promotion [45].

#### 3.3.3. Morphology Study of Char Residue

**Figure 9:**
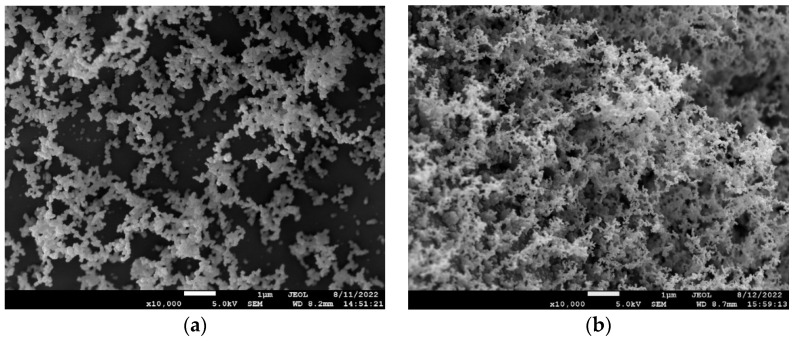
FESEM image of char residue: (**a**) Pure EP (**b**) EP/**4b**.

The morphology of the residual chars after the LOI test were studied, which present an important understanding of the flame-retardant effect of the additives on the EP matrix. Figure 9 represents the FESEM image of the char residue of pure EP (a) and EP/**4b** (b), with the highest LOI value.

The observed numerous open-holes in EP (Figure 9a) indicates the loose structure of the char residue. These open-holes allow the releases of combustible gas and promotes the burning rate. The aim of the addition of flame-retardant is to break the combustion cycle by reducing the factors of combustion such as combustible gas, heat, and oxygen. Char formation is one of the main elements of a flame-retardant mechanism, as they act as physical barriers to protect the matrix [46,47]. The denser and more compact char residue can be seen (Figure 9b) after the addition of compound **4b,** and this corresponded to the char yield in the TGA result, where this compound had a 12.33% higher yield compared to EP. The FESEM images prove that compound **4b** helped strengthen the formation of a homogenous char residue, leading to excellent flame-retardant properties.

### 3.4. Effect of Hexasusbstituted Cyclotriphosphazene Derivatives on the Mechanical Behavior

**Figure 10:**
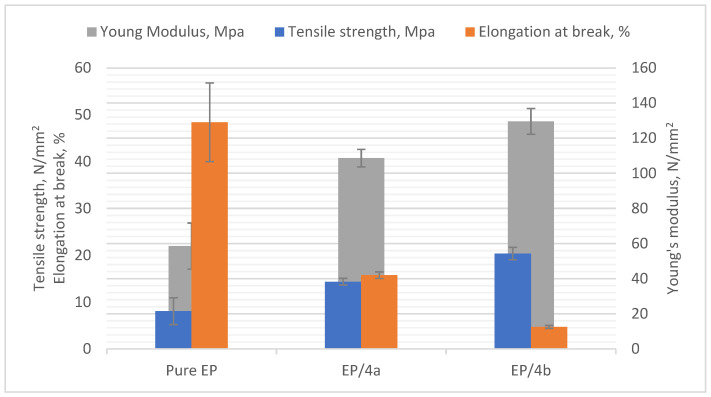
Mechanical properties of EP, and EP with FR additives.

Figure 10 illustrated the mechanical properties including tensile strength, elongation at break and Young’s modulus of EP and EP with FR additives. The trend shows that both tensile strength and Young’s modulus were increased with the addition of compounds **4a** and **4b**. However, the elongation has a different trend, with a decreasing value.

The tensile strength and Young’s modulus showed an increasing value for EP < **4a** < **4b**. The tensile strength went from 8.06 N/mm^2^ (pure EP) to 14.36 and 20.37 N/mm^2^ and the Young’s modulus went from 58.55 N/mm^2^ for pure EP to 108.67 and 129.58 N/mm^2^ for EP incorporated with compounds **4a** and **4b**, respectively. These phenomena were influenced by the increasing molecular weight and cross-linking attributed to the bulk compound and alkyl chain length of the additives [32]. The higher the tensile strength, the higher the ability of a material to bear maximum load, and the higher the Young’s modulus, the harder the material was to bend or deform.

The elongation at break gives information on the capability of a material to resist changes in shape [48]. Pure EP has a high elongation at break of 48.40%. In comparison, the elongation decreased drastically when 1 wt% of FR additives was added; 15.74 and 4.71% for compounds **4a** and **4b**, respectively. The high content of inorganic particles somehow affects the deterioration of flexibility when added to a polymer matrix [49]. Overall, these mechanical properties express the ability of a material to bear maximum load before breaking, the elasticity of a material, and the compatibility of the FR additives with the EP.

## 4. Conclusions

Halogen-free hexasubstituted cyclotriphosphazene compounds were synthesized and characterized using Fourier-transform infrared spectroscopy (FT-IR), and ^1^H, ^13^C, and ^31^P nuclear magnetic resonance (NMR) and CHN elemental analysis. A series of flame-retardant additives with Schiff-based compounds and different terminal alkyl chain lengths, labelled **4a** and **4b**, were prepared, and 1wt.% of these compounds was incorporated into epoxy resin as a polymer matrix to further investigate their fire-retardant properties and mechanical behavior. The flame-retardant properties were studied using thermogravimetric analysis (TGA) and the limiting oxygen index (LOI) test, and the char residue was analyzed under field emission scanning electron microscopy (FESEM), resulting in positive feedback with the highest LOI value shown in the EP incorporated with compound **4b** (26.71%), which increased by 17% from pure EP (22.75%). The mechanical behavior was evaluated using tensile strength, and it is fascinating that the FR additives were slightly affected by the addition. It shows an increment with a trend of EP < **4a** < **4b**. The tensile strength went from 8.06 N/mm^2^ (pure EP) to 14.36, and 20.37 N/mm^2^ indicates that the additives were compatible with epoxy resin.

## Figures and Tables

**Table 1:** Typical properties of epoxy resin and HCCP.

**Properties of Epoxy Resin** [34]	
Appearance	Clear liquid
Specific gravity at 25 °C	1.12 gm/cc
Viscosity at 25 °C	550 centipoises
Tensile strength	6.9 MPa
**Properties of Phosphonitrilic Chloride Trimer, HCCP (SDS, Sigma-Aldrich)**	
Appearance	Crystalline/light gray powder
Formula	Cl_6_N_3_P_3_
Molecular weight	347.66 g/mol
Melting point	112–115 °C
Initial boiling point and boiling range	127 °C at 17 hPa
Relative density at 25 °C	1.98 g/mL

**Table 2:** CHN elemental analysis of compounds **1b**, ****2a**–**b****, **3a**, and ****4a**–**b****.

Compound	Calculated (%)			Found (%)		
	C	H	N	C	H	N
C_21_H_34_O_2_, **1b**	79.19	10.76	-	78.84	10.67	-
C_19_H_32_N_2_O, **2a**	74.95	10.59	9.20	74.83	10.47	9.11
C_21_H_36_N_2_O, **2b**	75.85	10.92	8.42	75.79	10.86	8.38
C_42_H_30_N_3_O_12_P_3_, **3**	58.55	3.51	4.88	58.30	3.45	4.80
C_156_H_210_N_15_O_12_P_3_, **4a**	72.61	8.20	8.14	72.55	8.16	8.09
C_168_H_234_N_15_O_12_P_3_, **4b**	73.41	8.58	7.64	73.38	8.54	7.61

**Table 3:** Thermal properties of EP and modified EP.

Sample	T_5%_ (°C)	T_20%_ (°C)	T_max_ (°C)	Char Yield at 600 °C (wt.%)
Pure EP	168.7	344.6	372.6	8.0
EP/**4a**	164.3	337.9	372.3	11.57
EP/**4b**	164.4	334.5	374.0	12.33

**Table 4:** LOI test results.

Sample	LOI Value (%)	Sample	LOI Value (%)
Pure EP	22.75 (±0.00)	EP/**3**	24.90 (±0.00)
EP/**2a**	23.42 (±0.00)	EP/**4a**	26.55 (±0.00)
EP/**2b**	23.53 (±0.00)	EP/**4b**	26.71 (±0.00)

## Data Availability

Not applicable.
